# Honours and the Nursing Profession

**Published:** 1916-01-29

**Authors:** Eleanor C. Barton


### THE EDITOR'S LETTER-BOX.

### Honours and the Nursing Profession.

*To the Editor of* The Hospital.

Territorial Force Nursing Service.

No. 3 General Hospital. County of London.
Miss Barton, Chelsea Infirmary,
Principal Matron. S.W.,
January 24, 1916.

Sir,—My attention has been called to the article in The Hospital on the award of the R.R.C. under the heading of "Honours and the Nursing Profession." As the whole criticism centres round my name, I would be glad if you will publish this note to say that I do not agree in the least with the criticism. Your leader-writer is overlooking the fact that the R.R.C. is given for military nursing, and not for Poor-Law work. The matron of the 3rd London Hospital, who was selected by His Majesty for the honour, has rendered invaluable service in organising and superintending the nursing of the 3rd London Hospital, and I am very pleased with His Majesty's choice.—Yours very truly,

Eleanor C. Barton.

[Miss Barton's letter is worthy of her high and deserved reputation. She, however, misses the point. The paper on which she writes sets forth that she is principal matron of the 3rd London General Hospital. Twenty-three matrons of the Territorial Force Nursing Service received the R.R.C., of whom seventeen were principal matrons. Miss Barton is the only principal matron representing the Poor-Law Nursing Service in the Territorial Force. For the reasons stated in our article last week the list of honours loses in authority and dignity by the omission of the representative of the Poor-Law Nursing Service within the Force. We are fully conscious of the invaluable services rendered by Miss Holden, the matron of the 3rd London General Hospital, on whom the R.R.C. has been conferred, but this well-deserved honour in no way affects the undoubted claims of the Principal Matron of No. 3 General Hospital to the R.R.C. for the reasons stated.—Ed. The Hospital.]

